# A first genetic map of date palm (*Phoenix dactylifera*) reveals long-range genome structure conservation in the palms

**DOI:** 10.1186/1471-2164-15-285

**Published:** 2014-04-15

**Authors:** Lisa S Mathew, Manuel Spannagl, Ameena Al-Malki, Binu George, Maria F Torres, Eman K Al-Dous, Eman K Al-Azwani, Emad Hussein, Sweety Mathew, Klaus FX Mayer, Yasmin Ali Mohamoud, Karsten Suhre, Joel A Malek

**Affiliations:** 1 Genomics Laboratory, Weill Cornell Medical College in Qatar, Qatar Foundation, Doha, Qatar; 2 MIPS/IBIS, Helmholtz Zentrum München, 85764 Neuherberg, Germany; 3 Biotechnolgy Centre, Ministry of Environment, Doha, Qatar; 4 Department of Genetic Medicine, Weill Cornell Medical College in Qatar, Qatar Foundation, Doha, Qatar; 5 Agricultural Affairs Department, Ministry of Environment, Doha, Qatar; 6 Weill Cornell Medical College in Qatar, Qatar Foundation, Doha, Qatar

**Keywords:** Sex chromosome, Genotyping by sequencing, Comparative genomics

## Abstract

**Background:**

The date palm is one of the oldest cultivated fruit trees. It is critical in many ways to cultures in arid lands by providing highly nutritious fruit while surviving extreme heat and environmental conditions. Despite its importance from antiquity, few genetic resources are available for improving the productivity and development of the dioecious date palm. To date there has been no genetic map and no sex chromosome has been identified.

**Results:**

Here we present the first genetic map for date palm and identify the putative date palm sex chromosome. We placed ~4000 markers on the map using nearly 1200 framework markers spanning a total of 1293 cM. We have integrated the genetic map, derived from the Khalas cultivar, with the draft genome and placed up to 19% of the draft genome sequence scaffolds onto linkage groups for the first time. This analysis revealed approximately ~1.9 cM/Mb on the map. Comparison of the date palm linkage groups revealed significant long-range synteny to oil palm. Analysis of the date palm sex-determination region suggests it is telomeric on linkage group 12 and recombination is not suppressed in the full chromosome.

**Conclusions:**

Based on a modified gentoyping-by-sequencing approach we have overcome challenges due to lack of genetic resources and provide the first genetic map for date palm. Combined with the recent draft genome sequence of the same cultivar, this resource offers a critical new tool for date palm biotechnology, palm comparative genomics and a better understanding of sex chromosome development in the palms.

## Background

The date palm (*Phoenix dactylifera L.*) is a critical crop tree in a large section of North Africa, the Middle East and South Asia. It is one of 3 commercially important palms (Arecaceae) that include the monoecious oil and coconut palms. The date palm is dioecious with separate male and female trees. Only the female bears the commercially important date fruit. Trees grown from seed require approximately 6–8 years to flower before gender can be determined and no sex chromosome has been identified [1]. Sex determination in plants is highly heterogeneous and sex chromosomes in the palms appear to have developed more than once offering an excellent model for studying this process [2,3].

Despite the clear importance of the date palm, few genetic resources exist mainly due to a long generation time of approximately 6 years. A breeding program began in the United States in the 1940’s resulted in backcrossed varieties that exhibited depressed breeding ability and other physiological problems [4]. Previous research into the number of chromosomes in date palm suggests it has 18 chromosome pairs (2n = 36) [5] though some evidence for alternate numbers have been presented [6,7]. Technological advances allowed us to generate a draft sequence of a commercial cultivar of date palm [8] that was recently improved on [9] and revealed a genome size of approximately 670 Mb. The draft sequence, while critical to understanding gene content and polymorphism, does not provide structural information of the genome. We were able to use the draft sequence to identify scaffolds containing polymorphisms segregating with date palm gender but other studies such as a search for selective sweeps and quantitative trait loci (QTL) would benefit from a genetic map.

Construction of a genetic map in the absence of defined genetic resources is challenging. Specifically, outcrossed pedigrees require the identification of sufficient polymorphic markers between the male and female parents to allow map construction [10]. While date palm females, which bears the fruit, are well defined by cultivar, the males are much less so as they are simply used for pollination. The introduction of genotyping-by-sequencing (GBS) was important in overcoming these challenges and provides a method for genotyping thousands of new markers in potentially hundreds of offspring [11,12]. Accuracy in the GBS data is critical in our case as errors would result in lack of linkage or highly inflated map distances. We have adapted the method to our requirements by using a reduced representation method of GBS. We employed this adapted approach as each marker needed to be accurately genotyped by high depth sequencing rather than summed over a region as by Andolfatto and colleagues [13,14]. Here we present a first genetic map for date palm and its comparison to the other commercially important palms.

## Results and discussion

### Genotyping

Using various genotype quality requirements (see Methods below) such as minimum coverage and alignment scores, a total of 64,783 Single Nucleotide Polymorphisms (SNPs) were considered as genotyped at high quality. Using this method, comparison of ‘genotypes between two Khalas control libraries carried separately through the entire process revealed a genotype accuracy rate of 99.4%. Our previous genome sequence and SNP data from the Khalas cultivar allowed detection of male parent specific SNPs where Khalas was known to be homozygous. These male parent-specific SNPs were used to segregate progeny by which male parent they derived from. Four major populations derived from the same female parent but different male parents were selected for use in the genetic map and contained 29, 24, 17, and 15 individuals.

We obtained 5873 markers that fit our quality requirements, showed similar segregation in the populations and were called in at least 53 of the 85 individuals genotyped. Markers that shared the same segregation pattern across all progeny were grouped and a single representative marker was selected from each group. This collapsing resulted in 1235 representative markers of which 1199 were placed on the framework map. Markers placed on the map represented as few as one marker (especially male/female segregating markers) and as many as 28 markers. The 1199 representative markers represent 3976 total markers and these derive from 2115 unique segregating parent/scaffold combinations. That is, a scaffold could share two markers but this was only allowed if the markers segregated in different parents. The mapped marker set derived from 1823 scaffolds and these scaffolds span a total of 74,079,588 bp or ~ 20% of the 380 Mb of sequence in the assembly.

### Genetic map features

**Figure 1:**
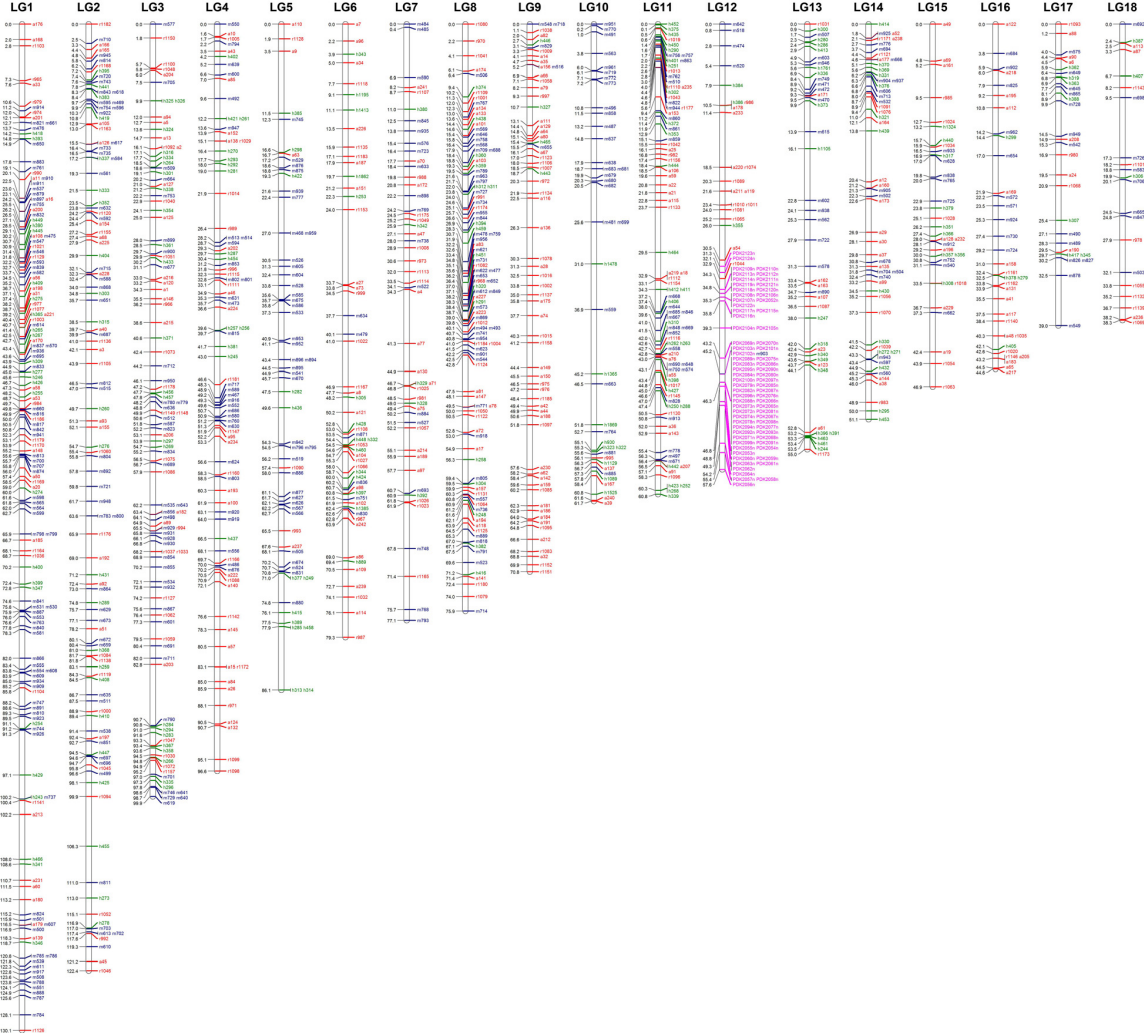
**A genetic map of date palm.** The consensus genetic map of a cross between Khalas female and 4 different male parents. Linkage groups were sorted by size and marker types were assigned colors. Red: heterozygous in female parent and homozygous in male parent; Green: heterozygous in both parents; Blue: heterozygous in the male parent and homozygous in the female parent; Purple: markers selected as linked to gender.

**Figure 2:**
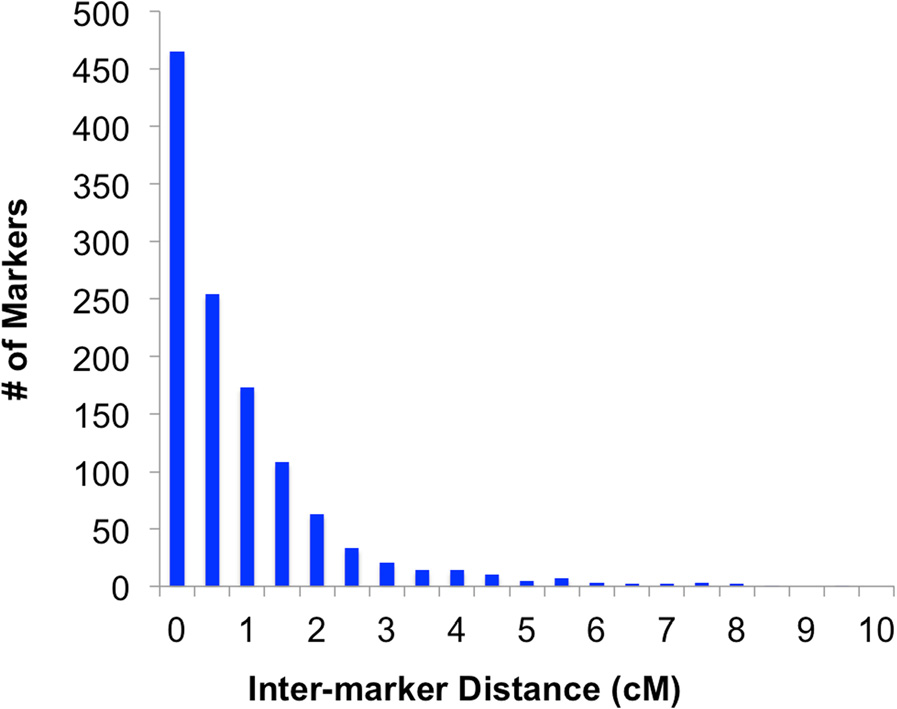
**Date palm genetic map Inter-marker distance plot.** The distance between adjacent markers on the genetic map were plotted as a histogram. A majority of markers are within 1 cM of each other, however, a tail of markers extends to 9.7 cM. This is likely due to bias of the method for certain gene dense regions as it relies on alignment to a reference sequence assembled from short read alignments.

The total genetic map length was 1293 centimorgans (cM) (Figure 1). Assuming a genome size of approximately 670 Mb leads to a genetic to physical distance conversion of approximately 1.93 cM/Mb of sequence or 520 kb/cM. Inter-marker distances were evenly distributed (Figure 2) though some biases were observed. The largest gap between any two markers was 9.7 cM on linkage group (LG) 6. Both LG5 and LG10 had an excess of gaps greater than 3 cM with a total of 9 and 7 respectively. Distribution of marker types among the linkage groups showed pronounced marker type bias in LG5, 6, 9, and 10. As an example, LG9 shows essentially no markers heterozygous in both parents or heterozygous in the male parent for 50 cM between cM 20 and the end of the linkage group. Long stretches on the map without certain marker types are likely due to not enough heterozygous markers genotyped in both parents. Markers heterozygous in both parents are necessary to integrate markers homozygous in only one parent into the map. Markers heterozygous in both parents were least likely to be captured given the requirement that they had to be heterozygous in all males used to create the mapping populations. Additionally, as the cross used for the map was taken from commercial cultivar, it is possible that there are indeed large stretches of homozygosity that decrease the chance for capturing those marker types. This both highlights the challenges of genetic mapping with outcrossed resources and the necessity of next-generation sequencing approaches to achieve high enough density to allow construction of a genetic map. The markers in the map derived from 1823 unique sequence scaffolds in the draft genome sequence. If each scaffold is considered to provide a single marker, this provides a unique marker every ~210 kb of the genome sequence.

**Figure 3:**
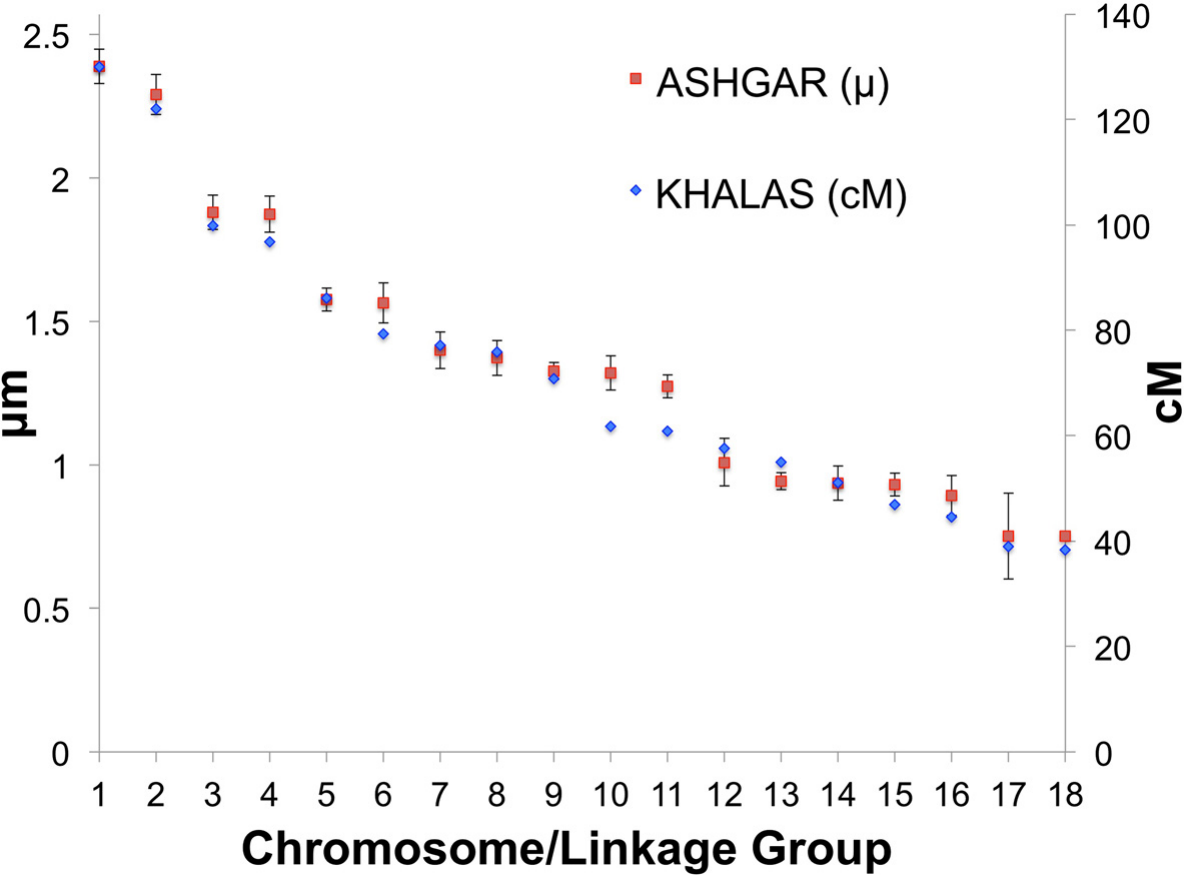
**Comparison of centimorgan lengths of linkage groups to physically measured date palm chromosomes.** Al-Salih and Al-Rawi [7] reported the chromosome lengths (μm) of the date palm Ashgar cultivar utilizing microscopy. We plotted their reported lengths and the centimorgan based lengths of linkage groups reported in this study for the Khalas cultivar. The scaled lengths from the two methods show very good correlation over all. Differences in lengths may be due to differences in cultivar type or incomplete extension of the linkage group from unincorporated markers.

The exact number of date palm chromosomes has been uncertain with some publications reporting 14, 16, or 18 pairs [6,7] with most evidence pointing to 18 pairs [5]. Our results agree well with Al Salih and Al-Rawi’s findings of both chromosome number and predicted length [7]. In their study of the Ashgar cultivar they detected 18 chromosome pairs and measured them with microscopy. To compare our genetic map we scaled lengths of our date palm linkage groups by adjusting the largest linkage group’s centimorgan length to their longest chromosome measured in micrometers (Figure 3). This revealed very good consistency between our genetic linkage group lengths to the physical lengths of the chromosomes. Indeed, only LG10 and 11 were significantly outside the standard deviation of physical measurements for the chromosomes. This discrepancy may be due to unplaced markers on the end of our linkage groups.

### Sex chromosome features

**Figure 4:**
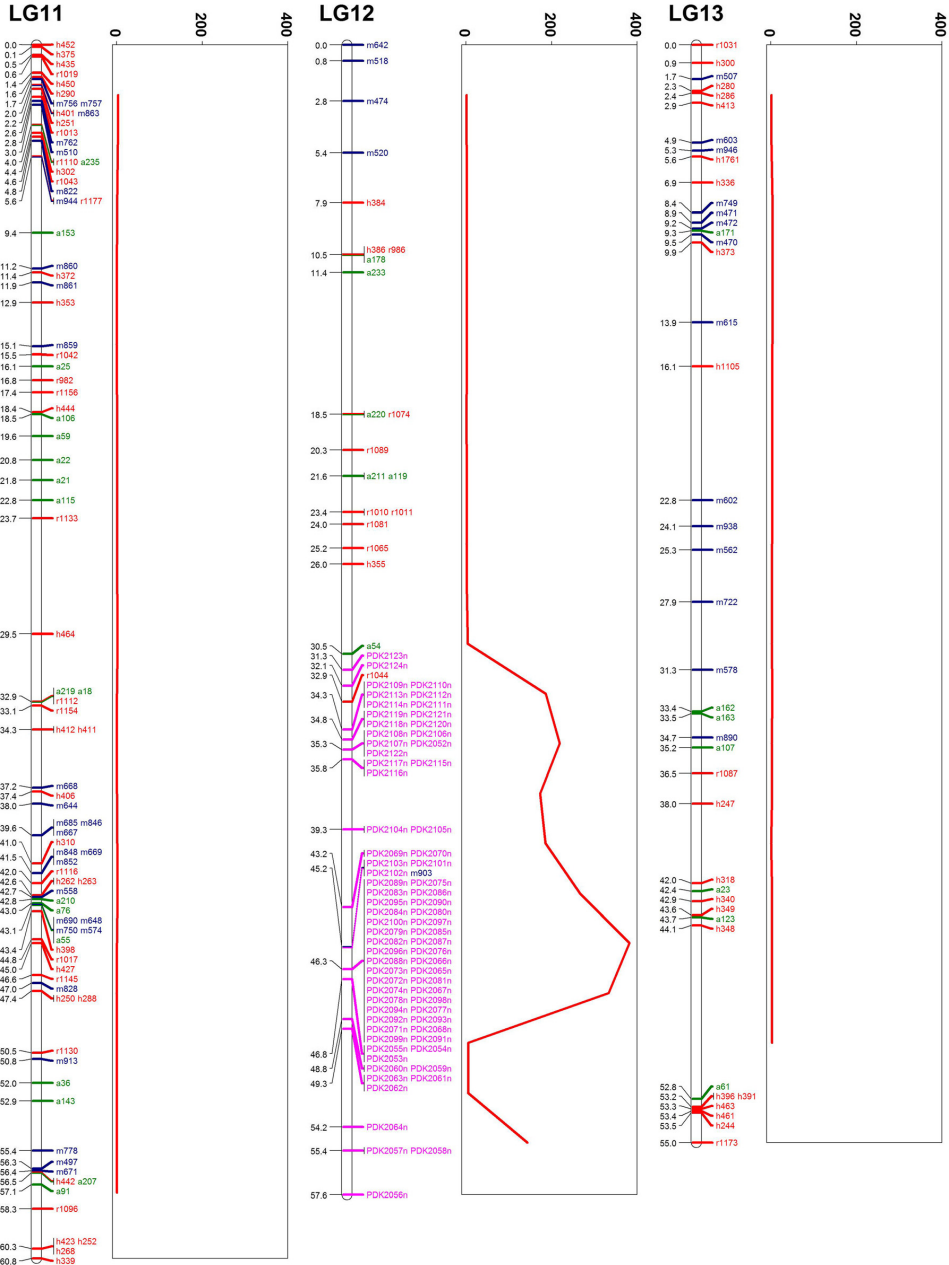
**Male/Female segregating single nucleotide polymorphism (SNP) density in selected linkage groups.** SNPs segregating with gender in 3 female and 2 male genomes were plotted. A 5 cM sliding window was used to determine the density of segregating SNPs per 100 kb and this was plotted as a red line next to the linkage group. LG12 contains the only region of high density segregating SNPs in the genome, suggesting it is indeed the sex chromosome. Localization of the sex determination region to a portion of the chromosome suggests the rest of the chromosome is still freely recombining.

**Figure 5:**
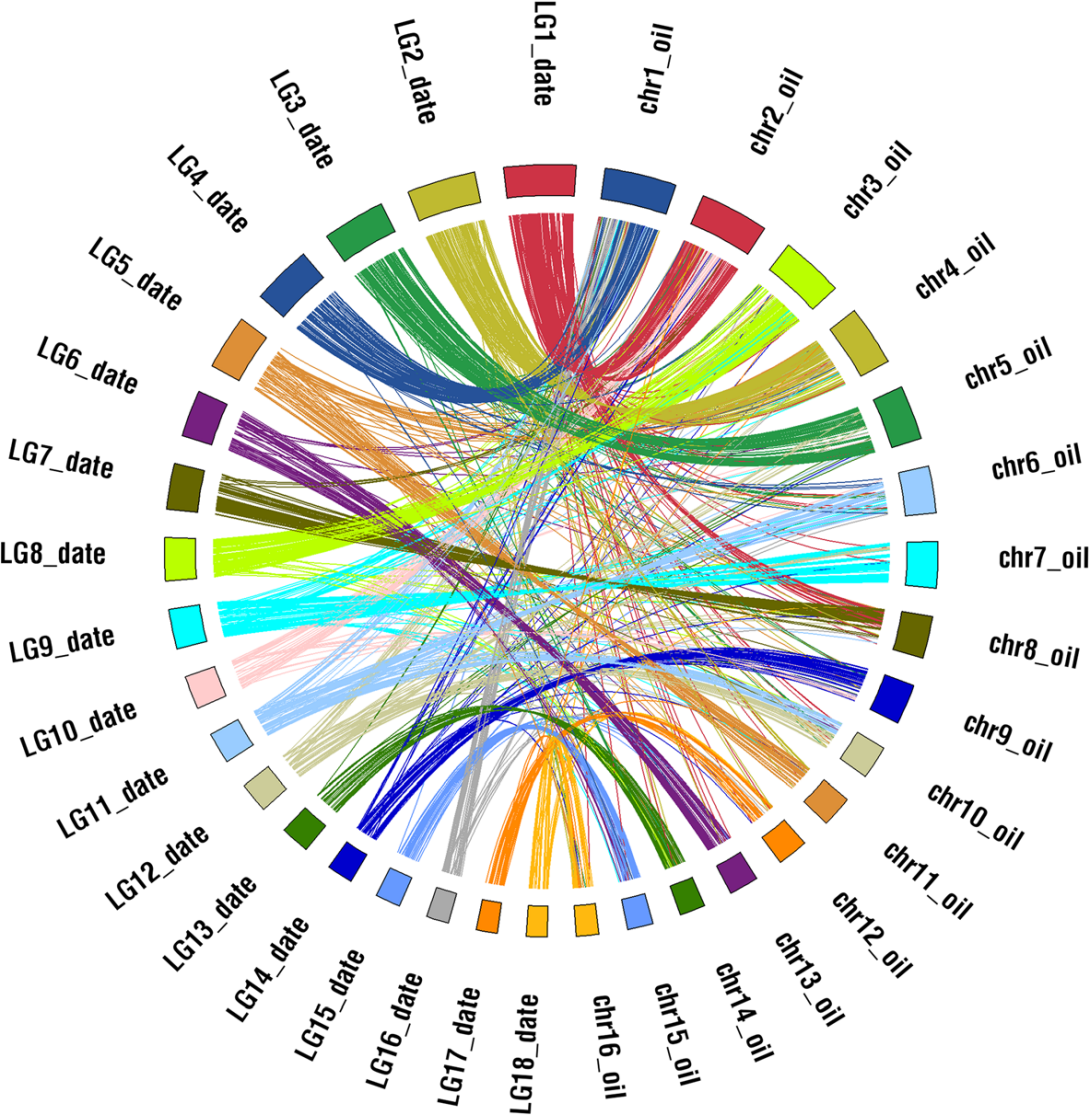
**Synteny between date palm genetic map and oil palm chromosomes.** Sequence surrounding markers from the date palm genetic map were aligned to oil palm chromosomes and locations reported. Linkage groups (LG) were colored by the most likely orthologous chromosome (chr) with orthology based on frequency of shared markers.

**Figure 6:**
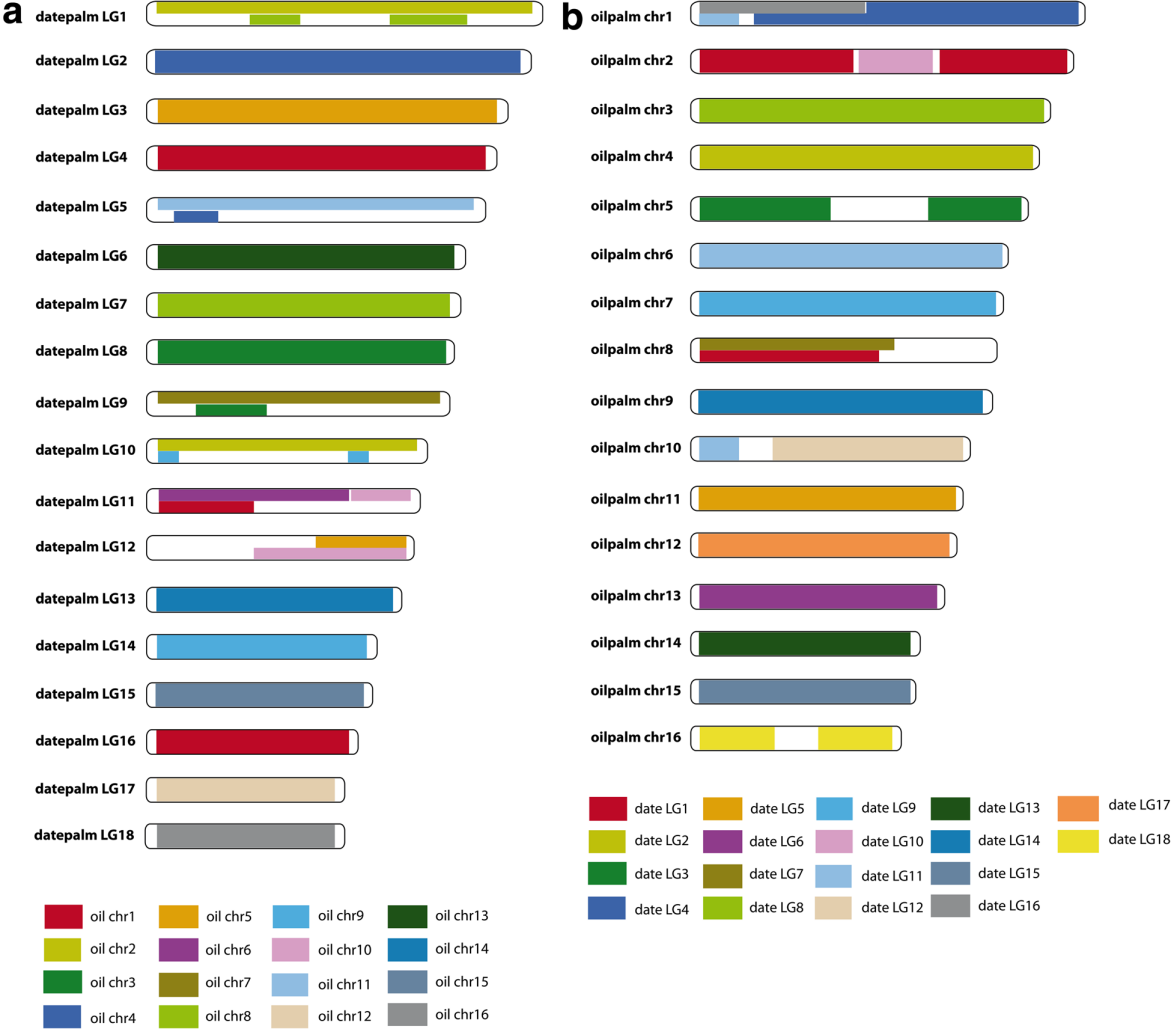
**Schematic overview of syntenically conserved blocks between the date palm genetic map and oil palm chromosome sequences as identified by CIRCOS plot in figure**5**.** Sequence surrounding markers from the date palm genetic map were aligned to oil palm chromosomes and locations reported on the **(a)** oil palm chromosomes (chr) or **(b)** date palm linkage groups (LG). Different color codes indicate syntenic blocks between the individual date palm linkage groups and corresponding regions on oil palm chromosomes as identified in CIRCOS Figures 5 and 7. In case of regions on oil palm chromosomes with indication of significant syntenic relationships to more than one distinct oil palm linkage group, split color bars were used. Areas on oil palm chromosomes with no support for any syntenic relation to date palm or showing promiscuous relations are colored in white.

Previous karyotypic analysis of the date palm genome has not identified a specific sex chromosome [1]. We observed that markers from scaffolds previously segregating with gender were all localized to the lower half of LG12 suggesting this may be the sex chromosome. We checked for the density of SNPs segregating with gender in all scaffolds and indeed the highest density was found in LG12 (Figure 4). This region segregating with gender is approximately 26 cM in length indicating the total physical length may be as long as 13 Mb or 2% of the genome. This is larger than we originally predicted [8] and may be due to the fact we utilized all markers in this region rather than collapsing to high-quality filtered representative markers as for the rest of the genome (see Methods). The use of non-quality filtered markers could lead to inclusion of genotyping errors and an inflation of the region’s size through a false increase in recombination frequency. Using markers from LG12 mapped to the more complete Saudi date palm genome (discussed below) scaffolds spanning 4.3 Mb were obtained. If the recent draft sequence is indeed ~82% complete, this would suggest the region is approximately 5.2 Mb. Additional work will be required to determine the exact non-recombining boundaries and would benefit from large clone sequencing such as bacterial artificial chromosome libraries. Comparison of the gender segregating region to the oil palm genome suggests that it may be rearranged in date palm. Indeed, markers from LG12 of date palm mostly mapped to Chr10 of oil palm though the sex determination region showed additional shorter regions of consecutive markers matching to oil palm chromosomes 1, 2 and 5 (Figures 5 and 6b). More detailed analysis at the DNA sequence level should shed light on why the date palm is dioecious despite sharing such high synteny and sequence similarity with the monoecious oil palm.

### Comparison to the recently updated draft sequence

Markers were mapped to the Saudi date palm genome [9] by BLAST [15] alignment of ~1000 bp surrounding the SNP. To be considered, BLAST alignments were required to be unique in the genome. 3884 (98%) of the marker regions uniquely aligned to 702 scaffolds in the Saudi date palm sequence. These 702 scaffolds span ~304 Mb (54%) of the 563.5 Mb assembled genome.

### High synteny in palm genome structure

To study palm genome structure, sequence surrounding markers on the genetic map were aligned to the recently released oil palm genome [16]. 2000 bp of sequence surrounding the markers were aligned to the oil palm scaffolds and oil palm chromosome locations were documented. It is clear from this comparison (Figure 5) that the two genomes maintain high levels of synteny. Indeed most of the 18 date palm linkage group were syntenic with one of the 16 oil palm chromosomes (Figure 6). How the two genomes diverged from 16 to 18 chromosomes is of interest. Based on telomere repeats detected in putative oil palm chromosome centromeres, it has been suggested that a robertsonian fusion caused the collapse of chromosomes into 2 and 14 in oil palm [16]. Our data supports this theory for oil palm chromosome 2. Synteny determined between the date palm genetic map and the oil palm chromosomes suggests that oil palm chromosome 2 constitutes a fusion of date palm chromosomes 1 and 10 whereas date palm LG11 shows blocks shared with oil palm chromosomes 1, 6 and 10. In addition, longer stretches on oil palm chromosome 1 correspond to regions on date palm chromosomes 4 and 16.

**Figure 7:**
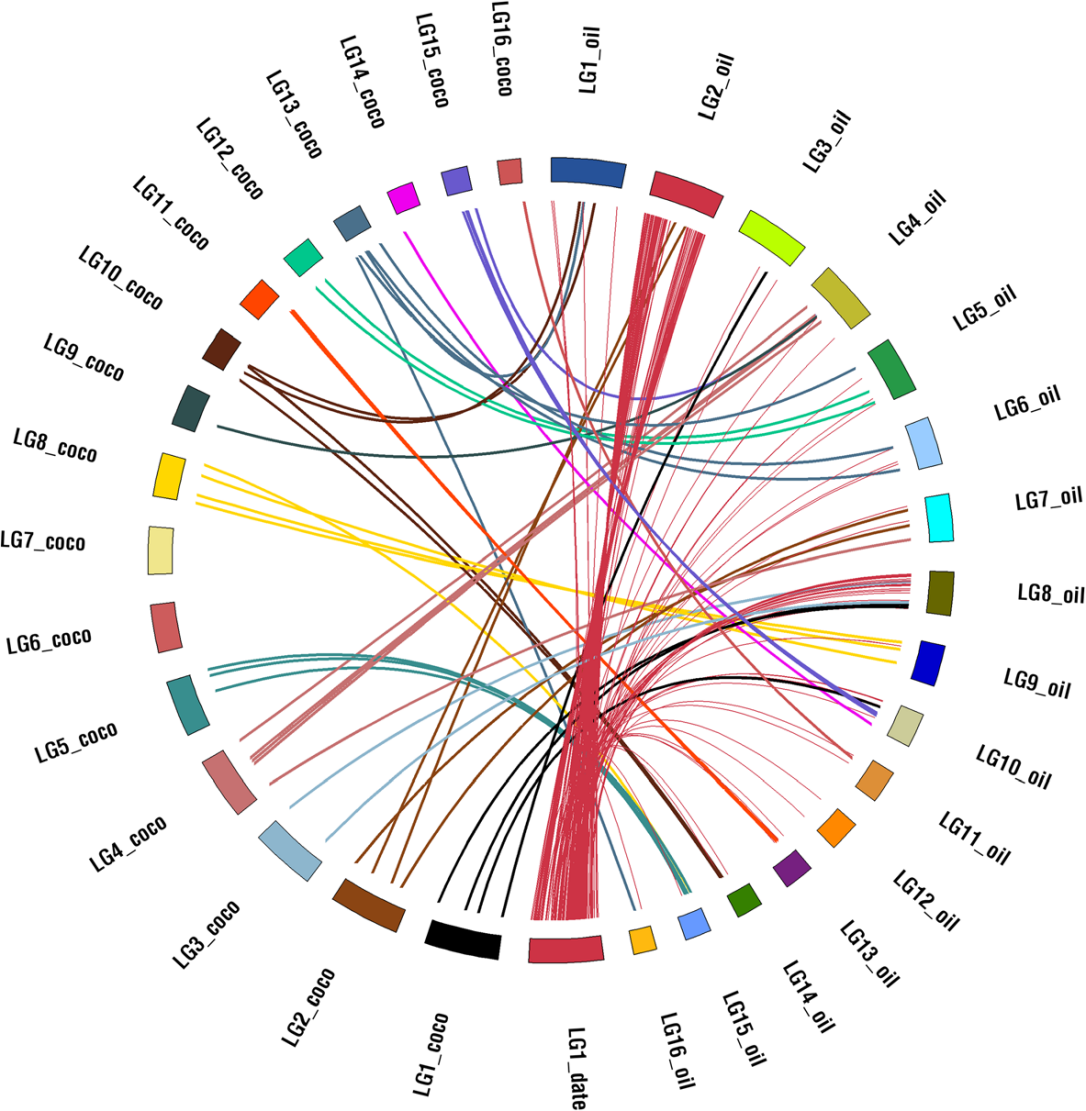
**Synteny between date palm genetic map linkage group 18, coconut sequence markers and oil palm chromosome sequences.** Similar to Figure 5 using coconut genetic map to compare to the oil palm. Coconut genetic map markers were mapped to scaffolds in the date palm genetic map. Linkage groups (LG) were colored by the most likely orthologous chromosome (chr) with orthology based on frequency of shared markers. Date palm LG1 is added for reference.

The same analysis approach was used to compare available sequence markers from the coconut genetic map [17,18] to the oil palm genome. While only 45 markers from the coconut map could be unambiguously aligned to the oil palm genome, a similar pattern appears with most markers from each coconut linkage group aligning to the same oil palm genome (Figure 7).

Despite the fact that the oil palm genome is more than double the size (1.535 Gb) of the date palm genome (0.67 Gb), long-range synteny is maintained and overall chromosome structure is very well preserved. It will be of interest to understand if the oil palm has added nearly double the sequence to the genome while maintaining chromosome structure or the date palm has lost ancestral DNA.

## Conclusions

We provide a first genetic map for the commercially important date palm tree and strong evidence for identifying the sex chromosome. Using next-generation sequencing we have overcome challenges due to lack of genetic resources for this tree. While markers on our map are not guaranteed to be polymorphic in other mapping populations, they nevertheless provide ordering of scaffolds for improved genome analysis. Additionally, by using SNPs from sequence scaffolds, other polymorphic markers within the same scaffolds could be selected for other populations. The high density of the markers on the map sets a target for future date palm genome sequence projects. Future research should aim for greater than 500 kb average scaffold length to ensure that most of the genome sequences will be properly ordered on the genetic map. To resolve more markers onto the map, it will be critical to expand these approaches with a larger pedigree (such as 200 offspring) derived from a single male and female parent. Extending the methods presented here will allow for an even denser genetic map of date palm and an improved resource for quantitative trait locus mapping.

For the first time we have localized the gender segregating region in date palm to LG12 suggesting this region may contain the sex determination genetic alteration. Using the genetic map we estimate the size of the region to be approximately 5 to 13 Mb and more work will be needed to ensure the true recombination boundaries. Identification of the gender segregating region will facilitate future studies on dioecy and the development of sex chromosomes. Indeed, our comparison of the region to the monoecious oil palm revealed shorter blocks of synteny in this region than the rest of the genome.

Analysis of the genetic map reveals important features in the date, oil and coconut palm genomes including maintenance of long range synteny despite dramatic genome size differences. Analysis of the genetic maps combined with genomic data will assist in the comparison and improvements of these 3 commercially important palms.

## Methods

### Genotyping-by-Sequencing

Date palm seeds were collected from a single Khalas cultivar individual at the agricultural affairs department farm in Rawdat Al-Faras, Al-Ghuwayriyah, Qatar. Seeds were washed and germinated at 29 C for 1 week and then transferred to potting soil. They were grown with minimal watering until primary leaves were ~15 cm. Leaf samples of 10 cm were collected for DNA extraction. Leaf material from each plant was arrayed into individual wells of 96-well plates, crushed with the TissueLyser II (Qiagen), and DNA extracted with the DNeasy 96 Plant Kit (Qiagen) according to the manufacturer’s protocol. As control for the full process, Khalas (female parent) leaves replaced two individuals in the 96 well plate. DNA was quantified and normalized using a Nanodrop (ThermoScientific). Genotyping-by-sequencing (GBS) libraries were constructed according to a modified protocol of Elshire and colleagues [11]. Samples were processed in batches of 24 to match barcodes available. Briefly, 150 ng of DNA per sample were digested with 1.25 units of ApeKI (New Engand Biolabs, USA) and then ligated to 10pmol of a universal PCR adapter and one of 24 possible barcoded adapters. DNA from the 24 samples, each with unique barcodes, was then pooled and cleaned with 1.2X Agencourt AMPure XP beads (Beckman Coulter, USA). To increase coverage of fewer SNPs and allow high quality calling of heterozygous alleles, a reduced-representation approach was used. Specifically, after pooling of 24 samples, the libraries were size selected on a 1.5% agarose gel from 350-550 bp using the automated Pippin Prep system (Sage Science, USA). Size selected samples were then amplified by PCR for 16 cycles and cleaned with a 1.2X Agencourt AMPure XP (Beckman Coulter, USA). Pools containing 24 libraries were sequenced on separate lanes of the HiSeq2000 (Illumina, USA) according to the manufacturer’s paired-end sequencing protocol. Five lanes of high-quality data representing 120 individuals were collected. Libraries were ‘spiked’ with 20% of a balanced nucleotide library to improve base-calling within the barcode.

DNA sequences were separated for each barcoded library, matched to the Date Palm V3 genome [8] using BOWTIE2 [19]. SNPs were then called with SAMTOOLS [20] to generate a VCF file. To ensure high-quality genotype calling, we required a SNP contain at least 15X coverage to be called. We required a SNP to be called in at least 80% of individuals to be considered. Individuals were then clustered in the Partek Genomics Suite (Partek, USA) using ~4000 SNPs that segregated in the male parent to determine their respective population. To detect the sex chromosome markers in the clustered data with similar inheritance patterns as markers in scaffolds previously shown to segregate with sex [8] were collected and added to the set of markers used to construct the map. That is, even single occurrence markers were used. While this may increase error rates in the region it provides higher numbers of mapped gender related scaffolds for future analysis.

### Genetic map construction

Utilizing the collected female parent genotype data we inferred male parent data by looking for 1:1 or 1:2:1 segregation patterns in the 4 populations. SNPs that shared segregation patterns in all populations were selected for use in the genetic map. Different markers having the same genotypes in all individuals were collapsed and the marker with the highest call-rate was selected as a representative. In the case of markers known to segregate male and female [8,21], we used all markers that clustered with markers on scaffolds known to segregate with sex (see above).

Genotypes were phased separately for each population and all populations merged using TMAP [22]. Two-points recombination data and LOD scores were calculated using CarthaGene [23]. Markers were grouped and ordered using JoinMap 4.0 [24]. Grouping was conducted with a LOD score of 10, the regression algorithm was used for marker ordering and the Kosambi function was used in marker distance calculation.

To study the sex control region, we utilized 5 genomes sequenced in our original study [8] including Khalas, Deglet Noor, Deglet Noor Backcross 5 male, Medjool, and Medjool Backcross 4 male. We documented SNPs that were homozygous in all females while heterozygous in both males. We then plotted the counts of these SNPs per 100 kb of scaffolds within a 5 cM sliding window and step size of 2.5 cM (Figure 4).

### Syntenic relationships between date palm genetic map and oil palm finished genome sequence

To determine syntenic relationships between the genetic map of *Phoenix dactylifera* (date palm) [8] and its close relative *Elaeis guineensis* (*pisifera* fruit form; oilpalm) we first downloaded the latest genome sequence release of oilpalm [16] from http://genomsawit.mpob.gov.my (16 EG5 chromosome sequences, without unanchored scaffolds) and extracted the position of 4850 distinct genetic markers from the datepalm genetic map (Additional file 1). From their position on a date palm sequence scaffold 1,000 bp upstream and downstream sequences were extracted for each marker where possible. In case of conflicts with a scaffold end or start we extracted sequence from the SNP until the beginning or end of the scaffold. BLASTN with an e-value cutoff of 10e-05 was used to identify homologous regions on the oil palm chromosomes for the 2000 bp marker-flanking date palm sequences. For a total of 4513 (out of 4850) date palm markers one or more homologous regions were found on the oil palm chromosome sequences; in case of multiple hits, only best blast hits (BBH) were considered for downstream analyses (in case of identical BBH bitscores all hits with this bitscore were considered).

The syntenic relationships established between date palm genetic map and oil palm chromosome sequences were plotted using the CIRCOS software [25] and are illustrated in Figure 5. Chromosomes with strong syntenic relations between date palm and oil palm were assigned the same color.

### Syntenic relationships between date palm genetic map, oil palm genome sequence and coconut genetic markers

To determine syntenic relationships between date palm, oil palm and coconut, markers from coconut were downloaded from GENBANK (http://www.ncbi.nlm.nih.gov) selecting only microsatellite DNA markers with significant (~500 bp) surrounding sequence. Location of the downloaded markers on the coconut genetic map was obtained from http://tropgenedb.cirad.fr/.

Fifty six coconut markers were associated with genomic sequence. BLASTN with an e-value cutoff of 10e-05 was used to identify homologous regions on the oil palm chromosomes for the coconut marker sequences. For a total of 45 (out of 56) coconut markers one or more homologous regions were found on the oil palm chromosome sequences; in case of multiple hits, only Best Blast Hits (BBH) were considered for downstream analyses (in case of identical BBH bitscores all hits with this bitscore were considered).

The syntenic relationships established between date palm genetic map, coconut markers and oil palm chromosome sequences were plotted individually for all 18 date palm linkage groups using the CIRCOS software [25] in Figure 7. Chromosomes with strong syntenic relations between date palm and coconut palm were assigned the same color.

## Availability of supporting data

The draft genome sequence used in this research is available at: http://qatar-weill.cornell.edu/research/datepalmGenome/download.html.

And at http://www.ncbi.nlm.nih.gov/genbank under the accession number: ACYX00000000.

## Competing interest

The authors declare no competing interests.

## Authors’ contributions

LSM extracted DNA, developed methods, constructed libraries, sequenced and assisted in analysis. MS conducted comparative genomic analysis. AA-M collected populations, grew plants, collected samples. BG conducted sequence processing and analysis. MFT conducted analysis of chromosome measurements. EA-D, EA-A conducted sequencing. EH helped conceive the project and select and collect mapping populations. SM collected samples and helped conceive project. KFXM, YAM and KS, helped conceive the project, interpret the data and write the manuscript. JAM conceived the project, designed experiments and wrote the manuscript. All authors read and approved the final manuscript.

## Supplementary Material

Additional file 1 **The date palm genetic map, representative markers and SNP marker locations.** Date Palm Genetic Map and Marker information. A spreadsheet containing all representative marker locations on the genetic map, the corresponding date palm sequence scaffold and location the SNP marker was derived from and all markers included in the representative group. Click here for file
